# Morphological and Performance Assessment of Commercial Menstrual and Incontinence Absorbent Hygiene Products

**DOI:** 10.3390/polym18030318

**Published:** 2026-01-24

**Authors:** Liesbeth Birchall, Millie Newmarch, Charles Cohen, Muhammad Tausif

**Affiliations:** 1 School of Design, University of Leeds, Leeds LS2 9JT, UK; l.l.birchall@leeds.ac.uk; 2 &Sisters Co., Ltd., London SW15 2SH, UK; millie@wearemooncup.com (M.N.); charlie@wearemooncup.com (C.C.)

**Keywords:** absorbent hygiene products, absorption, nonwovens, superabsorbent polymers, menstruation, incontinence

## Abstract

Disposable absorbent hygiene products (AHPs) contain plastics that are challenging to recycle and not biodegradable, making a significant contribution to landfill. Decreasing the nonbiodegradable mass of products could reduce this burden. Despite this, public data on how AHP design and material selection relate to performance is limited. In this work, fifteen commercial AHPs were characterised using dimensional measurement, infrared spectroscopy, and imaging. Simulated urination, air permeability, and moisture management testing were used to assess expected leakage and user comfort. Sustainable materials currently in use were identified, and their performance compared to typical plastics, informing opportunities to replace or reduce nonbiodegradable materials. Polybutylene adipate terephthalate-based leakproof layers replaced polyolefins. Commercial alternatives to polyacrylate superabsorbent polymers (SAPs), with comparable absorption, were not seen. Although absorbency correlated with the mass of absorbants, SAPs reduced surface moisture after absorption and are known for high absorption capacity under pressure, preventing rewetting. Channels and side guards were observed to prevent side leakage and guide fluid distribution, potentially reducing the need for nonbiodegradable nonwoven and absorbant content by promoting efficient use of the full product mass. While synthetic nonwovens typically outperformed cellulosics, apertured and layered nonwovens were associated with improved moisture transport; polylactic acid rivalled typical thermoplastics as a bio-derived, compostable alternative. Although the need for biopolymer-based SAPs and foams remains, it is hoped that these findings will guide AHP design and promote research in sustainable materials.

## 1. Introduction

Absorbent hygiene products (AHPs), such as tampons, diapers, and menstrual and incontinence pads, have become ubiquitous for convenient and dignified management of bodily fluids. However, their environmental impact is significant, as the majority of commercial products and their packaging are single-use and contain nonbiodegradable plastics. Menstruation affects approximately 43% of the population, according to UK Census data [1], while the prevalence of incontinence is typically reported as 25–45% of adults [2]. Considering menstruation management alone, it is estimated that 49 billion and 19 billion single-use menstrual products are consumed annually in the EU and USA, respectively [3]. It is estimated that 45,000 megatons of AHP waste are generated worldwide each year, as of 2019 [4].

Single-use AHPs and their packaging may consist of up to 90% fossil fuel-derived plastics [3] and make a substantial contribution to plastic waste accumulation. End-of-life management of AHPs is challenging and waste is typically incinerated or landfilled [4], where plastics and superabsorbent polymers (SAPs) from AHPs are reported to take up to 500 years to degrade [5]. Materials from AHPs enter and contaminate aquatic environments, where pollutants and microplastics can bioaccumulate and cause ecological harm [6,7]. Additionally, production of AHPs is reliant on fossil fuels and generates pollutants and greenhouse gases, which contribute to climate change [8]. Given the rate at which these disposable products are consumed, there is an urgent need for sustainable design of AHPs.

AHPs with a pad structure are widely used for the management of incontinence and menstruation and act as disposable inserts for undergarments. Menstrual pads must act as effective barriers and prevent migration of menses, which would stain clothing. Per cycle, the median mass of menses from pad-weighing studies is 56.7 g and the mean mass is 77.6 g [9]. Menstrual pads do not need as high an absorption rate as incontinence pads, as menses are ejected gradually over several days rather than in discrete events. In contrast, incontinence typically has a greater fluid volume, depending on severity, and the risk of leakage increases with volume [10]. Using the Incontinence Severity Index (ISI), pad-weighing tests have shown that the average leakage per 24 h is 39 g for moderate, 100 g for severe, and 200 g for very severe incontinence [11]. Urination flow rates follow a parabolic curve and reach a typical maximum of 20–30 mL·s^−1^, meaning that rapid absorption is needed to prevent run-off [12]. Despite differences in composition, viscosity, and pH between menses and urine, menstrual pads are used to manage incontinence. Disposable menstrual pads have been shown to be suitable for mild incontinence, although disposable incontinence pads were preferred by users: menstrual pads absorbed an average of 11.1 g, with a leakage rate of 26.0%, while light incontinence pads absorbed 17.0 g, with a leakage rate of 15.7% [10].

**Figure 1:**
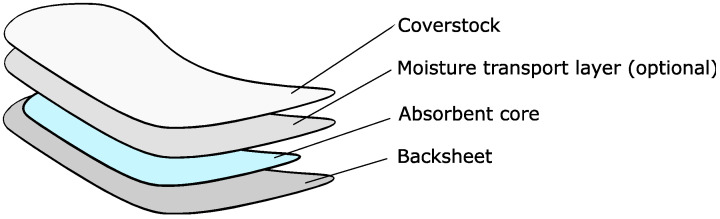
Diagram of a generic absorbent hygiene product.

AHP pads have a generic, layered structure, as shown below in Figure 1. Coverstocks are nonwoven materials that act as the interface between a user and a product; to ensure user comfort, they must be soft and wick moisture away from the skin. The pad may contain a nonwoven moisture transport layer underneath the coverstock to further direct fluids: acquisition/distribution layers (ADLs) lie above the core and enhance fluid transport from the product surface to the core, while core wraps enclose the core for structural reinforcement. The core consists of absorbent materials—such as wood pulp fluff, cellulosic card wadding, and superabsorbent polymers (SAPs)—which act to securely contain liquids. Beneath this, the product backsheet is a leakproof layer which prevents fluids from leaking and is attached to the user’s clothing using adhesive. Outside of these essential features, fastening and shaping can be controlled using wings, elastic, and side strips to further prevent leakage. Structure and design features are known to impact performance and user ratings [10].

Despite the wide scope of the problem, there is a lack of comprehensive data on AHP functionality. Published reports have favoured clinical studies of products in use, but analysis of products themselves has been limited. A review of the AHP literature informs that clinical studies have been limited by small study size and lack of statistical power and that a paucity of evidence and numerous research gaps remain [10,13]. Without well-considered study designs, such research can fail to identify the product features that contribute to perceived performance and user preferences. Older comparative studies become outdated as products are taken off the market, particularly if detailed description and understanding of product features were not provided.

Outside of clinical studies, much research has focused on development and/or analysis of AHP components, rather than holistic study. Skin health associated with AHP use is a key issue, and development of comfortable nonwovens has been explored. Edwards et al. prepared 50:50 griege cotton/polypropylene hydroentangled nonwovens with fluid handling performance consistent with polypropylene top sheets typically used in AHPs [14]. Kaplan et al. investigated the antibacterial properties and air permeability of polypropylene top sheets treated with antibacterial agents [15]. Atasağun and Kara performed a study on moisture management and frictional properties of top sheets, testing a combination of three polypropylene coverstocks with and without three different ADLs [16]. However, nonwovens in the literature lack structural features that are common in commercial AHPs, such as embossing, aperturing, and elastic elements. The range of materials explored is also typically limited to polypropylene and/or cotton. Recent research is focused on the development of absorbents or AHPs from apparent sustainable fibre sources such as agave [17], bamboo [18], banana [19], corn husk [20], pineapple [21], and textile waste [22]. Assessment of product performance may be limited: absorption capacity assessments in the literature have typically relied on the overall absorption capacity of an AHP determined by the Rothwell measurement, which neglects structural aspects of leakage from body-worn AHPs [23]. While some comparison is made to commercial AHPs, research is often disembodied from the commercial product landscape and comparative analysis of product design is limited. Panjwani et al. recently published a comprehensive review of biodegradable materials for AHPs [24]. In their overview of commercial biodegradable AHPs, there remains a clear lack of analysis of such products and their performance in the literature. To the best of the authors’ knowledge, there is no data available on the comparative performance of commercial AHPs made from a range of materials/technologies. A deeper understanding of how materials and product features lead to functionality is necessary for the development of the next generation of sustainable or high-performance AHPs.

In this paper, AHP pads were examined, deconstructed, and assessed for key performance metrics to gain information on aspects of commercial design. A range of commercial products are described thoroughly to give a holistic study of design and functionality, focusing on the management of incontinence. This novel data will help to understand the performance of key products and highlight opportunities for the use of sustainable materials in AHPs.

## 2. Materials and Methods

Fifteen commercial adult absorbent hygiene products (AHPs) were selected: three to reflect market-leading products, and other products marketed as innovative, environmentally friendly products. Seven incontinence pads and eight menstrual pads were investigated, all of which were disposable, self-adhesive undergarment inserts. These were labelled by function (I or M for incontinence or menstrual), then numbered were assigned by grouping products by coverstock material and then in order of increasing product mass. Incontinence products weighing under 11 g were observed to market themselves as light or medium capacity, and those over 14 g as heavy. Two sets of products shared a brand: products I1 and I2, and products I4 and M4. Products I2 and M2–5 were marketed as night products. Products and product components were unfolded and conditioned under a standard atmosphere (20 °C, 65% relative humidity) according to ISO 139:2005 [25] for 24 h prior to all work to standardise the moisture content of the fabrics.

Products were weighed and measured using a precision balance and ruler, respectively, before dissection. The wings and edges were then cut off to enable separation of products into layers, which were similarly weighed and measured. Thickness of the products and nonwovens was measured according to ISO 9073-2:1997 [26] using a ProGage thickness gauge (Thwing-Albert Instruments, West Berlin, NJ, USA), where the nonwoven thickness is taken as the distance between two plates when the sample is under a uniform pressure of 0.5 kPa. The weight and thickness of release papers for adhesives was measured separately and subtracted. At the product level, fabric mass areal density (g·m^−2^) was calculated by measuring the mass of individual component layers and employing image analysis to measure the area of the respective layers.

Fourier transform infrared attenuated total reflectance (FTIR-ATR) spectroscopy was used to match component spectra to known materials against a library database. Spectra were collected as an average of 100 scans between 4000 and 550 cm^−1^ using a Spectrum 3 spectrometer (Perkin Elmer, Shelton, CT, USA) fitted with a golden gate ATR (Specac Ltd., Orpington, UK) at a scan rate of 0.5 cm s^−1^ with 0.4 cm^−1^ resolution.

Scanning electron microscopy (SEM) and optical microscopy were used to collect images of product components to identify component materials, such as morphology and fabric structures. A M205 C microscope (Leica, Wetzlar, Germany) was used for lower magnification imaging of nonwovens and to estimate fibre diameter (assuming circular cross-sections). Fibre diameter measurement was performed at 12.5× magnification using the line tool in ImageJ 2.14.0/0.154f. Three micrographs were taken per nonwoven sample and five measurements were taken from each for a total of fifteen measurements per fibre. Samples for SEM were sputter-coated in gold (20 nm) and imaged at 10 kV using a EVO MA15 microscope (Zeiss, Oberkochen, Germany). Particle sizing of SAPs was performed in ImageJ by thresholding electron micrographs, and diameters of spherical particles in clusters were measured as described above for fibre diameter. Nonwoven structures were suggested based on visual inspection and optical microscopy.

**Figure 2:**
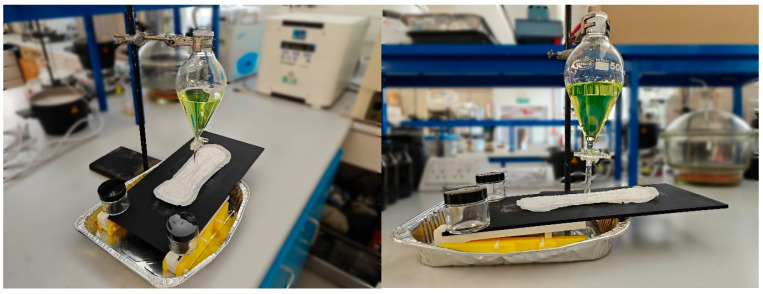
Simulated urination apparatus. The background has been blurred for clarity.

Urination onto products was simulated using an in-house rig and artificial urine. The experimental set up, Figure 2, comprised a recording device with an adjustable stand, a separatory funnel to dispense test fluid dosages, an acrylic stage on a 3D-printed mount, and an overspill tray. The stage was held at a 5° angle to allow fluid to run off while also allowing time for absorption. Products were secured to the acrylic test stage using the product adhesive and synthetic urine dispensed onto the centre of the pad. The failure mechanisms of pads were observed, and the pad and stage were weighed before and after dispensing to calculate the volume of liquid held.

Pad-weighing experiments have shown that the average leakage per 24 h was ≤51 g for moderate, 75–128 g for severe, and 131–268 g for very severe incontinence [11]. Typical human urination events are reported to take between 17.5 and 30.7 s on average [27], within the 21 ± 1 s value reported for mammals with >3 kg body mass [28]. Therefore, urination was simulated with separatory funnels capable of dispensing target volumes within this timeframe. Test dosages of 50, 100, and 250 mL were selected to approximate light, medium, and heavy incontinence capacities.

Incontinence pads were tested in triplicate using the highest dosage at which fluid leakage was observed: either 250 mL dispensed over 20 s or 100 mL dispensed over 31 s. Menstrual pads were tested by dispensing 50 mL over 16 s.

Artificial urine was prepared using a formulation based on ISO 20696 [29], which is used to test sterile urethral catheters for single use, as listed in Table 1 below. The final concentration was reduced to two-thirds that of the initial ISO 20696 formulation to better match the midpoint of the range of human urine concentration [30]. All chemicals were sourced from Sigma Aldrich (Merck, Darmstadt, Germany) and used as received. Fluorescein was added as a dye to aid visualisation of fluid dynamics (2 µM concentration in artificial urine), and all chemicals were dissolved in distilled water together to 1 L in a volumetric flask.

The breathability of top sheets was assessed using air permeability testing. Nonwovens above the absorbent core of products were removed and tested. Top sheets consisting of multiple nonwoven layers were tested as one piece to better reflect the product in use. All measurements were taken from the centre of products and performed in triplicate. Air permeability measurements were taken according to the NWSP 070.1.R0 (20) standard [31] using an air permeability tester (TexTest AG, Schwerzenbach, Switzerland), testing air flow through the nonwoven sample with a 20 cm^2^ test area and 100 Pa pressure. Specific permeability was then calculated according to Darcy’s Law (Equation (1)):

(1) $$q=\;\frac{k\Delta p}{\eta h}$$

where *q* is specific permeability (m^2^), *k* is air permeability (m/s), *η* is the viscosity of air (Pa s) at 20 °C and 1 bar, *h* is thickness of the material (m), and Δ*p* is pressure drop (Pa).

Fluid transport in top sheets was assessed using moisture management testing (MMT). An M290 MMT (SDL Atlas, Rock Hill, SC, USA) fitted with two sets of concentric electrode sensors was used to take conductance-based measurements of the top and bottom surfaces of fabric samples. Top sheets were dry-ironed flat on a low heat setting, re-conditioned according to ISO 139:2005 [25], and then cut to size to fit the electrodes. A saline solution of 16 ± 0.2 mS conductivity (approx. 0.9 wt%) was dispensed on the sample from above, and parameters of fluid transport were obtained, according to AATCC TM195-2011 (Liquid Moisture Management Properties of Textile Fabrics) [32]. One-way moisture transport quantified the directionality of flow from the top surface to the bottom, and the overall moisture management coefficient (OMMC) summarised the efficiency of both vertical and lateral wicking.

## 3. Results and Discussion

### 3.1. Material Composition

The materials and construction of the fifteen selected products are shown below in Table 2, comparing the dimensions and components of each commercial AHP pad.

#### 3.1.1. Dimensional Properties

**Figure 3:**
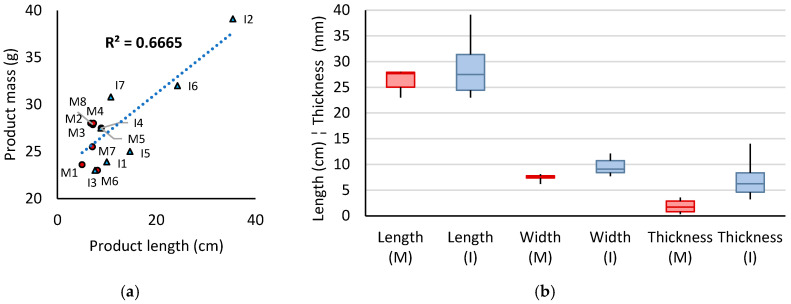
(**a**) Product length plotted against product mass; menstrual and incontinence products are plotted with circular and triangular markers, respectively. The dotted line shows the linear trendline between product length and product mass. Grey leader lines are used to aid labelling of data points. (**b**) Box plot of dimensions of AHPs, where M denotes menstrual (red) and I incontinence (blue) products. Whiskers indicate the range of values.

A summary of the dimensional data of whole products is given below in Figure 3. Pads varied in size: 23–39 cm for length, 6–12 cm for width, and 1.6–14.0 mm for thickness. These dimensions positively correlated with product weight (0.67 R^2^ for length), which ranged from 5.0 to 35.4 g. Understandably, higher target absorption volume was associated with larger dimensions, to accommodate the greater mass of absorbent material required. Incontinence pads were more variable in size than menstrual pads due to variations in degrees of incontinence. The average product weight was 15.9 g for incontinence and 7.2 g for menstrual. Self-reported light–medium incontinence products weighed <11 g and heavy incontinence >14 g.

Differences in target absorption between and within use cases is reflected in the data. Incontinence pads were near-universally thicker than menstrual pads, with an average thickness of 7.1 mm compared to 1.9 mm in menstrual products. This reflects the greater volumes and flow rates in incontinence, where a greater core mass is needed to immobilise and absorb liquid.

Product width was the least variable dimension as pads must fit within the spacing between the user’s limbs at the groin, as marked by the two inguinal creases, for stable positioning on an undergarment gusset. Despite this intercrural limit, incontinence products were wider than menstrual, with 9.6 cm and 7.5 cm averages, respectively. The maximum measured width was 12.1 cm in product I2; its effective width during use is narrower, as this is an elasticated product which was flattened for measurement.

Lastly, the shortest and median lengths of AHPs were near-identical regardless of use case, but incontinence pads had greater variation in length, with a maximum of 39.1 cm (I2). Products that intended to absorb greater volumes of fluid were longer: this can help to prevent leakage by contouring around the pelvis and to achieve a suitable core mass without an exorbitant thickness. The average length was 28.8 cm for incontinence products and 26.4 cm for menstrual.

**Figure 4:**
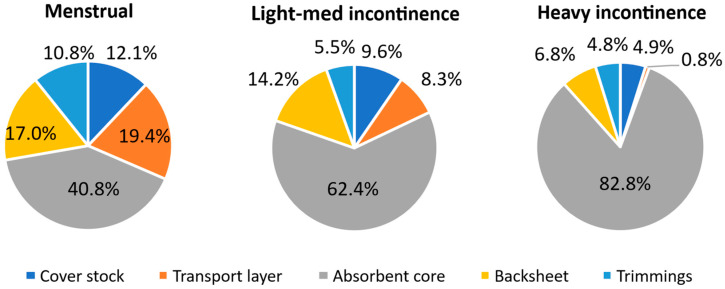
Composition of commercial pad AHPs by use case.

To better understand the components within the product, the layers comprising the AHPs were separated for further analysis. The average composition of products by mass is shown below in Figure 4 for menstrual products (excluding M5), light to medium incontinence, and heavy incontinence. Constituents have been grouped into top sheets (coverstock and moisture transport layers), absorbent core (fluff, cellulose wadding, and/or biopolymer granules), backsheet, and trimmings (wings and the margin cut around the edge of the pad in order to separate the layers).

The percentage of absorbents by mass increased as the overall product weight increased and the target absorption increased: the absorbent core of menstrual products accounted for 40.8% of its weight (2.8 g of 7.0 g), compared to 62.5% in light–medium incontinence products (5.8 g of 9.3 g), and 82.8% in heavy incontinence products (20.5 g of 24.7 g). The menstrual products contained a lower percentage of absorbents than the EDANA data for ultrathin sanitary pads, 54% absorbent core [33]; this may be due to the prevalence of winged products in this dataset. Across all incontinence products, the absorbent core represented 76.0% of the product mass, in good agreement with EDANA data for all-in-one incontinence products, 79% absorbents [34].

In menstrual products, the top sheets weighed almost twice as much as the backsheet, whereas in incontinence products, they were similar in mass. While the average mass of coverstock was close between use cases (0.84 g menstrual and 0.93 g incontinence), most of the incontinence products lacked a moisture transport layer compared to only a single menstrual product M6. There was a greater prevalence of ADLs in incontinence products and core wraps in menstrual products. The increased use of core wraps in menstrual products likely represents a greater need to reinforce thin pads and to consolidate the smaller absorbent core during production [35]. In contrast, incontinence pads (excluding I4) contained a greater volume of fluff pulp and so thinner top sheets may be preferred.

Significant design differences were observed between the two use cases: all menstrual products had wings, compared to only one incontinence product. Menstrual products have a lower absorption capacity requirement and therefore require less material than incontinence products, allowing them to be lighter and thinner. Wings are widely used to help fasten the product to undergarments and provide a barrier against staining [35]. The average wingspan observed was 15 cm, double that of the average width; this reflects the maximum wing length for folded wings to share a single release paper.

For incontinence products, the higher volumes and flow rates result in increased liquid travel and a more significant risk of leakage as compared to menstruation. Therefore, a greater design focus on product shaping to direct and contain fluids was observed. Incontinence products were seen to near-universally contain fluff in the absorbent core, compared to under half of the menstrual products. Fluff is used to confer bulk to the product, which better enables the product to contour to the body and optimises fluid flow [36]. Structures along the length of the pad were used to control liquid movement. In products I3–4, side strips of nonwovens were seen on top of the coverstock on each side of the absorbent core to prevent fluid run-off at the product edges [37,38]. The majority of incontinence products (I2, I4–6) used lateral elastic in coverstock to shape the pad. This results in curved products that contour around the body and direct fluid into the centre [39]. The width of incontinence pads was greater than for menstrual, to give fuller coverage, accommodate more absorbents, and/or create a gutter-like shape to prevent side leakage. In contrast, wings were seen only in product I4: wings can pull downwardly on AHPs, leading to an unacceptable risk of side leakage [38]. Overall, appropriate product structuring and mass of absorbents is vital to use case.

#### 3.1.2. Nonwoven Fabrics

Average fibre diameters in coverstock varied from 12.4 to 30.9 µm, with an overall average of 18.9 ± 5.9 µm standard deviation. Across products, natural fibres had larger diameters than synthetic fibres and greater variation in measurement due to their lower circularity (approx. 23.2 ± 6.0 µm vs. 15.9 ± 3.4 µm). Coarser natural fibres were seen in the transport layers than in coverstock (23.2 µm vs. 32.1 µm), as transport layers do not come into direct contact with skin and therefore do not need to be as soft.

Coverstocks are reported to be between 10 and 30 g·m^−2^ for heavy incontinence products and up to 70 g·m^−2^ for light to moderate incontinence pads [35]. However, a significant difference between use cases was not observed in this dataset. Areal density did correlate with the structure of top sheets, where products containing multiple nonwoven layers had less dense coverstock. The average density of coverstock was 58.2 g·m^−2^ in single-layer products, 42.5 g·m^−2^ in products with a core wrap, and 24.6 g·m^−2^ in products with an ADL.

Spunbond polypropylene (PP) is commonly used in AHPs [35] and was present in the market-leading products I1, I2, and M1. While the hydrophobicity of PP keeps the surface dry, this prevents wetting of the filament surface; the PP is kept thin and porous so that liquid passes through the material efficiently. The average density of polypropylene coverstock was approximately half that of other materials (24.6 g·m^−2^ vs. 49.7 g·m^−2^). Polyolefins are predominantly derived from petrochemicals and have minimal inherent biodegradability, with negligible degradation in soil using ISO 14852 [40,41,42]. While recycling of PP and PE is well-established and may offset the environmental impact, municipal recycling of absorbent hygiene products themselves is not typically feasible due to the challenge of separating the various materials. Only 0.3% of global diaper waste is recycled [5]; the typical fate of PP or PE in used AHPs is landfill or incineration [4]. It may be prudent to make greater use of biodegradable polymers until recycling of AHP waste is more commonplace. While reusable products benefit from the material longevity of polyolefins, and recycling is reported to be the AHP waste management process with the lowest carbon emissions, human toxicity impact, and cumulative energy demand [4], disposable products, which are rarely recycled, do not benefit from recycling, and the accumulation of nonbiodegradable polymers is environmentally detrimental. Excluding recycling processes, AHP polymers are incinerated or placed in landfill, either directly as sterilised fluff or as waste following biological waste treatment. AHPs with biodegradable polymers could be compatible with the above processes and reduce accumulation in landfill and environmental contamination. Fully biodegradable AHPs would be ideal for potential biological waste treatment of AHP waste, which is estimated to have lower human toxicity impact and lower cumulative energy demand than mechanical–thermal conversion into sterilised fluff or the baseline (incineration/landfill), albeit higher CO_2_ emissions than mechanical–thermal conversion [4]. Ideally, materials which readily biodegrade in a range of environments could ensure that products break down, even when waste collection or sorting is poor, and could be revalorised by home composting.

Established synthetics may be substituted for bioplastics due to their processability, renewable feedstocks and/or improved biodegradability, albeit at a higher cost. Product I3 was unique in its use of spunbond polylactic acid (PLA), a thermoplastic increasingly used in compostable nonwovens for its processability, mechanical properties, and relatively low cost [43,44]. PLA monomers are typically derived from maize; microbial fermentation of plant waste streams is another potential source, though commercially viable processes are still in development [45,46]. However, while PLA is industrially compostable, PLA degrades slowly (1% rate in soil, ISO 14852) [40,42] and has negligible degradation in marine environments, and efficient degradation requires higher temperatures than typically achieved during home composting [44].

Other bioplastics with more rapid biodegradation than PLA have been suggested for use in nonwovens, including polyhydroxyalkanoates (PHAs), polyester amide (PEA), and the petrochemically derived polycaprolactone (PCL) [42,43,47]. Unfortunately, as these bioplastics are more expensive and their nonwoven processing less established, they are largely limited to research and niche medical applications at present. PHAs are of particular interest for their biocompatibility and high degradation in water, soil, and composting environments [44]; poly(3-hydroxybutyrate-co-3-hydroxyvalerate (PHBV) had a degradation rate of 53% in soil (ISO 14852), twice that of cellulose [40,42]. While PHAs are comparatively expensive, Nodax^®^ is a PHA reportedly used in flushable nonwovens [43], and production of PHAs is expected to increase [48]. Should PHAs become more economically viable, their use in nonwovens may be promising.

Regenerated cellulose was the dominant plant-derived fibre and was seen in hydroentangled (spunlace) viscose nonwovens in I4 and M2–5. Fibres are typically spun from wood or bamboo pulp dopes, though cellulose is the most abundant natural polymer and plant or textile waste may be valorised as a source of regenerated cellulose [49,50]. Unmodified regenerated cellulose has comparable biodegradation to cellulose [43,51]. However, the environmental impact of fibre production must be considered: the Viscose process uses chemicals which are harmful to the environment, while the Lyocell process uses the non-toxic solvent NMMO, of which >99% is recovered [52]. Lyocell is typically more expensive than cotton or viscose; 80% of regenerated cellulose produced is viscose while only 4% is Lyocell [53]. Additionally, cellulose is thermoset and must be processed and bonded as fibres, whereas melt-processed nonwovens are associated with higher production rates and lower costs because they are produced directly from molten feedstock [54].

Cotton was the most common natural fibre used, found in hydroentangled coverstock in products I5–7, M4, and M7–8. However, challenges in satisfying the global demand for cotton and in its environmental impact have driven demand for alternative natural fibres for nonwovens; cotton agriculture typically requires intensive irrigation and uses a high volume of pesticides [43,47]. Alternative fibres which require minimal water, require minimal pesticides, do not harm soil health, grow quickly, can be grown locally relative to the point of manufacture or processing, and/or require minimal processing are desirable.

Product M6 used an unspecified natural fibre with a diameter of 30.9 µm, in combination with a water-soluble fibre as coverstock in a flushable menstrual pad. Product M5 used hemp in their coverstock, a fast-growing and sustainable crop [55]. Compared to cotton, hemp fibres are coarser and undergo more complex extraction processes. In M5, hemp fibre bundles were much coarser than other fibres seen in commercial products, necessitating their carding with viscose to result in finer fibres for perceived softness. The selection of a natural fibre should consider agricultural sustainability, supply chains, and fibre processing, as well as fibre properties which influence the tactile properties of the nonwoven. Sustainable production of natural fibre nonwovens with acceptable strength, density, and softness is challenging; a comprehensive review of bio-based fibres and binders for nonwovens is given by Santos et al. [43].

The materials and construction of the fifteen selected products are shown below in Table 3, comparing the material selection, processing, and fibres of each coverstock and holistic properties of the top sheets (excluding side guards). Thickness and areal density values are given for the product centre. Nonwoven classes are suggested based on visual inspection and optical microscopy. 

For moisture transport layers, ADLs were seen in products with polypropylene coverstock (I1–2 and M1), while core wrapping was seen predominantly in menstrual products and in products with viscose coverstock (I3–4, M2–4, and M7–8). ADLs may be required to enhance moisture transport across hydrophobic PP coverstock, while core wraps may be used in thinner products to reinforce the core, which prevents SAPs from making pinholes in nonwovens or films when under compression by continuous drum systems [35]. M8 used a bilayered nonwoven cotton to act as both coverstock and core wrap.

**Figure 5:**
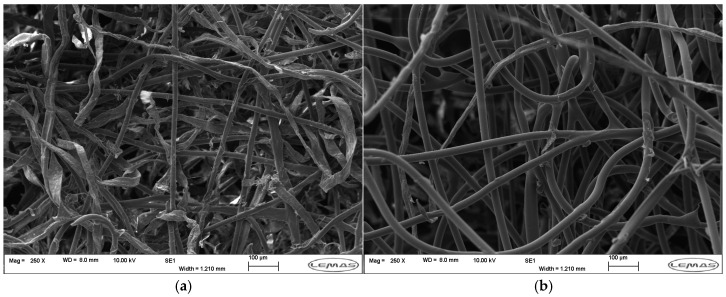
SEM images of (**a**) 56.6 g·m^−2^ PE/cotton core wrap from product M4; (**b**) 65.6 g·m^−2^ synthetic polymer ADL from product I2.

Three main categories of transport layer materials were seen in the product dataset. Airlaid transport layers, shown below in Figure 5, universally had higher density than the corresponding coverstock (average core wrap 55.1 g·m^−2^ and ADL 77.0 g·m^−2^). This likely results in sufficient capacity in the transport layer for efficient transport from the surface. M1–M4 contained a synthetic polymer—typically polyethylene (PE)—bonded with cotton. Products I1–2 had fully synthetic ADLs consisting of PP and a polyester or polyacrylate. Lastly, products I3 and products M7–8, which are marketed as biodegradable, used cellulosics to wrap the core. As with coverstock materials, there may be an opportunity to replace thermoplastics with bioplastics and cotton with other cellulosic fibres.

Lastly, strategies to structurally optimise the top sheets were observed. In both product use cases, coverstocks were modified using aperturing or calendering to create pores in natural fibre nonwovens (I7 and M6–8) and to induce point bonding in synthetic nonwovens (I1–3 and M1), respectively. In products I5–6, there was a variation in fibre density along longitudinal rows of the coverstock created by rows of waterjets during the hydroentangling process. All of the above alter local density, which can alter local fluid transport. Apertures in products may improve appearance, softness, penetration, and rewet [56].

Embossing of dotted ovals in top sheets was seen in products I3–4, M1–5, and M7–8. This may be used to create channels in pads to control fluid movement, to bond layers, and to structure the pad to better conform to the user, reducing side leakage [57]. Embossing was seen in thin pads with less fluff and the presence of a core wrap, suggesting that this approach was typically used to further structure thin pads. Lateral elastic was used to structure incontinence pads (I2 and I4–6), creating a barrier to side leakage. Clancy and Malone-Lee observed that an elasticated coverstock was associated with a slight reduction in leakage for urine volumes under 200 mL [58]. While synthetic elastomers are petrochemically derived and nonbiodegradable, there is a drive towards natural rubber and bio-derived monomers, although cost and scalability are barriers to their usage [59]. Laminate nonwoven structures have also been explored as a route to elastic-like coverstock [60].

A summary of materials and structural properties of the transport layers in the fifteen products are shown below in Table 4, divided into ADLs and then core wraps.

#### 3.1.3. Absorbent Core

All products contained cellulosic absorbent material in their core. Two-thirds of products contained fluff while one-third contained cellulosic wadding; product I7 was the only one to contain both fluff and wadding. The typical fluff is pine Kraft pulp, which can absorb 12 g water per gram under free swell but only 2 g/g under 5 kPa pressure [35,36]. Products I5–6 and M7–8 used cotton fluff; while this has greater absorbency (19 g/g), there can be concerns about the sustainability depending on cotton cultivation practices [36,43]. Alternative natural fibres can be used to produce sustainably farmed fluff, particularly in arid regions where wood feedstocks are limited [17]. Alternative or modified fluffs with improved absorption are known, but the reduction in product thickness must outweigh the increased cost to the consumer [35]. Similar material considerations apply to the wadding, which is typically cardstock. A comprehensive review of cellulosic absorbents is given in Hubbe et al. [36]. Regardless, fluff and wadding are unable to rival the absorption of superabsorbent polymers, and products with SAPs have been seen to have lower rates of leakage than those without [10].

Superabsorbent polymers are cross-linked polymers able to absorb and hold up to 1000 times their own weight in water and are typically sodium polyacrylate-based [61]. Two-thirds of products contained SAPs in one of two core structures: layered products, where SAP and fluff were sandwiched in cellulose wadding (I4, I7, and M2–4), and blended products, where SAP granules were distributed through airlaid wood pulp fluff (I1–3, I5–6, and M1). Product I2 contained two layers of airlaid fluff; double-core structures have been correlated with reduced leakage [58]. Menstrual products tended to be layered and incontinence blended. All products without fluff contained a core wrap to reinforce the absorbent core during production [35].

**Figure 6:**
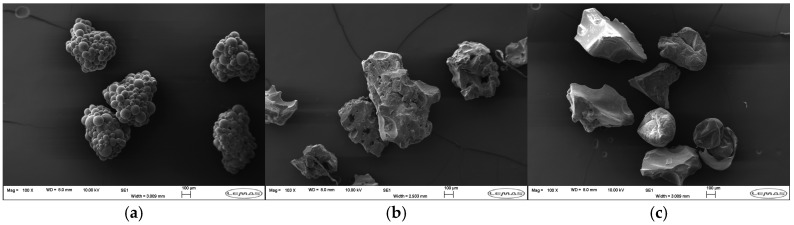
SEM micrographs of SAP granules from commercial absorbent hygiene products. (**a**) Product I3; (**b**) Product I1; (**c**) Product I6.

SEM micrographs of SAPs isolated from products are shown below in Figure 6. SAP granules varied in size, with an overall average Feret diameter of 582 ± 187 µm (standard deviation). Products I3–4 and M2–4 had SAPs consisting of clusters of perfect spheres; there was a broad range of sphere diameters in all products, approximately 5–150 µm. The spherical morphology suggests that an inverse suspension polymerisation is used, where an aqueous solution of monomers is stirred to obtain a water-in-oil emulsion. Products I1–2 had jagged, angular, and porous SAPs. The irregular shape implies shear planes, suggesting that this SAP was produced by bulk polymerisation and then granularised. Lastly, I6 showed two morphologies and may be a blend of materials: jagged particles with smooth surfaces are likely from a bulk polymerisation process followed by granularisation, albeit with lower porosity than I1–2, while particles resembling dehydrated microspheres may be from a separate solution phase polymerisation.

Given that SAPs are network polymers with a high density of hydrophilic groups along the polymer chain, it was not simple to identify the base polymers of the SAPs. As polyacrylate is petrochemically derived and nonbiodegradable, a reduction in the acrylate content of SAPs is beneficial. Unfortunately, it was not determined whether products used bulk polyacrylic acid (PAA), starch-co-acrylic grafted polymers, or modified biodegradable polymers. While research into bio-sourced and biodegradable SAPs is ongoing [61], to the best of our knowledge, there are no commercial, fully biodegradable SAPs.

Products which did not contain SAPs used cellulosic absorbents: I7 used wood pulp fluff and cellulose wadding, M7–8 used cotton fluff alone, and M5 used a folded sheet of chemically bonded hemp fibre. Hemp is a low-cost, sustainable natural fibre; the lumen gives fibres a high surface area for water sorption [55]. Product M6 was unique in using carboxymethylcellulose (CMC) granules as an absorbent, a hydrophilic modified cellulose which forms hydrogels [36]. As the CMC in M6 was not cross-linked, it was able to fully dissolve and the flushable product able to disintegrate in water. Modified biopolymers can rapidly gel but are limited to ≤10 g/g absorbency unless crosslinked to form SAPs [36,61].

A summary of the absorbants present in the commercial products is given below in Table 5.

Further development of biodegradable absorbents with performance rivalling that of synthetic SAPs is needed. In addition to biopolymer SAPs and hydrogels, cellulose foams have been proposed for rapid absorption with good mechanical properties [62,63,64]. While cellulose foams were not observed in the selected commercial products, they are an active area of absorbent materials research.

Deville et al. investigated the use of lignocellulosic foam as an alternative to fluff in menstrual products [62]. Thermomechanical bamboo pulp was mixed in water and a non-ionic polyglucoside surfactant was added, stirring at 2000 rpm for 13 min in total. This was poured into moulds, water was allowed to drain out through a 100 µm filter for one hour, and then foams were dried at 40 °C for 12 h. The foam had a free swell capacity of 22.5 g water/g foam, exceeding that of a selected commercial pad, but a water retention of 50% under 5 kPa load. While the foam had lower absorption capacity than commercial SAPs themselves, it outperformed typical fluff and could potentially replace or reduce the need for fluff–SAP blends for target performance.

A key barrier to the commercialisation of nanocellulosic foams is the cost of production. Patiño-Masó et al. produced cellulose nanofibers (CNFs) via oxidation of Kraft pulp using sodium hypochlorite with a low loading of TEMPO catalyst (2 g/kg) to reduce cost [64]. CFCs were dispersed in water using high-pressure homogenisation and then freeze-dried. CFC foams showed rapid absorption and had greater or equal free swell absorption capacity to the commercial SAP in 0.9% saline (56.8–90 g/g compared to approx. 58 g/g). CFC foams were produced with higher absorption capacity under pressure than the free swell absorption capacity of a commercial diaper, with estimated costs between EUR 0.04 and 0.06/diaper.

In brief, cellulosic foams have potential to outperform contemporary absorbents, and there is ongoing development into potential routes to economical, mass-produced absorbent cellulosic foams.

#### 3.1.4. Backsheet

Backsheet polymers were identified using FTIR spectroscopy. Five products were found to contain a polyethylene (PE) backsheet, the typical material for the industry [35]. In products I2, I5, and M3, a broad peak at around 1407 cm^−1^ and peaks at 872 and 713 cm^−1^ indicated the presence of calcium carbonate filler [65], which is used at around 50 wt% to make breathable PE backsheets [35].

Product M3 marketed its backsheet as plant-based and its product as biodegradable in 3 months. Bio-PE is chemically identical to PE from petrochemical sources, with the same limited biodegradability, but presents a renewable source. Based on tonnage data from market reports [66,67,68], only 0.3% of PE in 2021 and 0.04% of PP in 2025 were biologically derived, and there are higher costs associated with biosynthetics compared to petrochemicals. The bio-PE film is likely oxo-degradable, where transition metal salts and other additives are typically added at 1–3 wt% to promote oxidative degradation of polymers into fragments which can be more easily biodegraded. However, this reduces polymer lifespan and recyclability, and generates microplastics which may persist in the environment [69]. There has been considerable debate on the biodegradability of oxo-degradable polymers, and Directive 2019/904 bans their use in the EU. Further validation of biodegradation in various environments is needed.

All other products used biodegradable polymers. In products I3 and M6, the backsheet consisted of a layer or coating on top of a nonwoven rather than a polymer film. In I3, this layer was used to adhere the tissue paper core wrap to the coverstock and to form a thick waterproof barrier on the nonwoven backsheet. In M6, the film was identified as starch or modified starch [70]. Carbonyl stretching modes, absent in unmodified starches, were observed at 1709 cm^−1^ and 1267 cm^−1^. Starch-based films are reported to have moisture sensitivity and limited mechanical strength [71]; these properties may favour this product’s disintegration in water under shear stress.

Biodegradable backsheets (excluding M6) contained polybutylene adipate terephthalate (PBAT)-based polymer films, as used in blends with PLA and/or starch to produce commercial compostable bags [72]. The presence of a broad peak at around 3300 cm^−1^ in products I3, I6 and I7 corresponds to an O-H stretch and implies a high proportion of starch in the blend [73]. From the FTIR spectra and the materials reported by AHP manufacturers, blends are tentatively suggested: I4, M2, and M4 are PLA/PBAT; I3, M5, and M7–8 are PLA/PBAT/starch; and I6–7 are PBAT/starch, or PLA/PBAT/starch with high starch content. PBAT-based backsheets are advantageous in that the material has a high technology readiness level, representing 13.4% of the biodegradable bioplastics market in 2021 [44]. Films are commercially available and are certified to biodegradation and composting standards. Transitioning from PE to PBAT-based films is feasible; the limiting aspect is cost.

Overall, a wide range of materials are used in AHPs, with manufacturers keen to meet demands for sustainability. Some opportunities are highlighted, but sustainability is multifaceted and challenging for companies to define or optimise. While recycling of polymers in AHPs presents the greatest reduction in carbon footprint in life cycle analyses [4], the lack of cost-effective processes to recycle laminate products means that material degradation remains a key focus. Transitioning to more sustainable materials is also not simple. Materials must be both cost-effective and trusted by consumers if they are to be implemented successfully. Therefore, the performance of the commercial AHPs were assessed in terms of absorption and user comfort metrics.

A summary of the backsheet materials and masses for each commercial AHP is given below in Table 6.

### 3.2. Absorption

Users rate the ability of a pad to withstand insult as the most important criteria of an absorbent hygiene product [10,74]. In order to obtain both qualitative and quantitative data on how AHPs manage fluid, urination was simulated using dosage volumes to test different incontinence severities. Set volumes of synthetic urine (Table 1) were dispensed onto pads adhered to a plate at a 5° angle. The fluid distributions, visible sheen on the top surfaces, and failure mechanisms of pads were filmed, and the pad and stage were weighed before and after dispensing to calculate the volume of liquid held. This comparative data is only valid for discussing performance of these menstrual products for the management of incontinence. While saline may simulate sweat or urine, menses differ in viscosity, pH, and composition. Further work with a suitable test fluid would be needed to assess absorption of menses.

**Figure 7:**
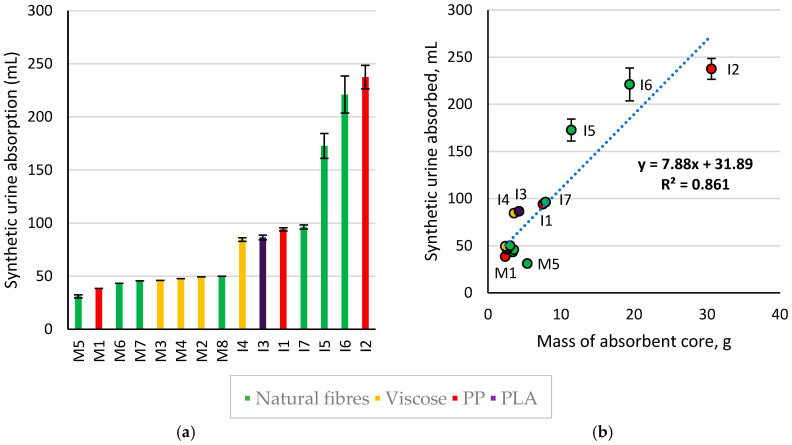
Volume of synthetic urine absorbed, plotted (**a**) for each product and (**b**) as a function of the mass of the absorbent core. The linear trendline is plotted as a dotted line. Error bars denote standard deviation. Colours indicate the material of the coverstock.

Absorption volumes for AHPs are shown below in Figure 7. Menstrual pads appeared effective for mild incontinence: the majority absorbed >45 mL, and M2 and M8 had minimal or no leakage, implying that they have higher absorbency than the 50 mL dosage. AHPs marketed as light–medium incontinence pads absorbed an average of 90.4 mL, while heavy pads were able to absorb 100 mL without leakage and absorbed between 173 and 238 mL of a 250 mL dosage.

The volume absorbed correlated with the mass of the absorbent core (0.86 R^2^). While the mass of the polymer or SAP granules was not quantified, the percentage of SAP in the core appeared to be associated with higher absorbency per unit mass at high loadings: I3 and I4 had visibly high SAP content and high absorbency for their weight. While I7 lacked SAPs, both I1 and I7 had a high proportion of fluff and therefore similar absorbency. The relation of absorbent core mass to absorption plateaued at I2, which appeared to use excess fluff for bulking as a night product. Lastly, M5 had the lowest absorbency (31 mL), using a chemically bonded hemp nonwoven rather than loose fluff pulp and having no absorbent polymer granules. This data is limited in that absorbants are considered as a whole. Further work to estimate the mass of polymer granules and fluff and to measure the absorption capacity of the SAPs in each product could gain insight into the separate contributions of absorbants. This may give insight into how to maximise the absorption efficiency of products and/or reduce the mass of fluff or SAPs.

**Figure 8:**
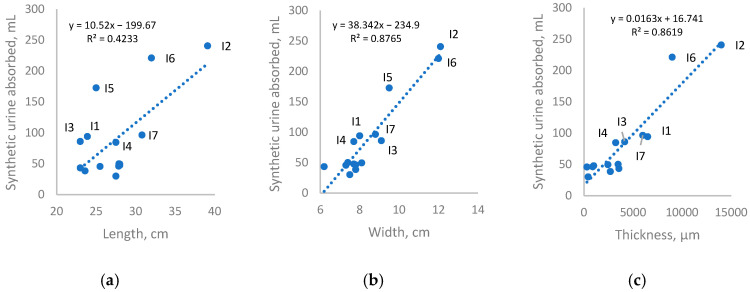
Volume of synthetic urine absorbed, plotted as a function of (**a**) AHP length; (**b**) AHP width; (**c**) and AHP thickness. Linear trendlines are plotted as dotted lines.

Amount of synthetic urine absorbed during testing also correlated with AHP dimensions, as shown in Figure 8. AHP dimensions correlated with mass of absorbants, as products needed to accommodate the volume of absorbants. With respect to AHPs for menstruation, light–medium incontinence, and heavy incontinence, products with higher target absorbencies had higher average widths and thicknesses, and greater experimental absorption. Larger dimensions may also present a slight physical barrier to fluid run-off, as fluid must travel further for leakage to occur, slowing flow and allowing more time for absorption to occur. It is unclear whether this had a significant impact in this study as this was coupled to mass of absorbants. While length having a weaker correlation to measured absorbency did coincide with the observed tendency for fluids to leak from sides rather than from the front, this was largely due to similar average lengths of AHPs for menstruation and for light–medium incontinence (26.4 cm and 26.3 cm, respectively).

Leakage predominantly occurred from sides of pads during testing. The swelling of SAPs in the product centre was noted to cause some pads to become convex as liquid was absorbed, increasing the likelihood of run-off. Side strips in products I3–4 were observed to reduce side leakage, as suggested in the patent literature [37,38]; instead, leakage occurred from the front of the pad due to fluid run-off. This allowed more time for SAPs to swell and resulted in their high absorbency relative to their core mass. The lateral elastic in products I2 and I4–6 could potentially contour to the user to reduce side leakage during wear [39,58]; however, the elastic was cut to make products lay flat for testing and this was not explored. The potential for wings to increase side leakage was similarly unexplored [38].

More even distribution of fluid through pads was noted in products with SAPs and with transport layers (ADLs or core wraps). Channels—whether embossed, apertured, or a variation in fibre density in longitudinal rows—were observed to direct or facilitate flow to a lesser extent. Embossed channels were observed to distribute fluid to the embossed points, in agreement with the patent literature [57]. No obvious differences in fluid distribution were seen between layered or blended SAPs, though the higher absorbency products in this dataset all favoured the latter approach. SAPs dispersed in fluff appeared to have different distributions in wet product cross-sections. I3 appeared to have a layer of granules on top of a dense layer of wet fluff, products I5–6 appeared to have SAP–fluff–SAP layers, and I2 appeared to have several alternating layers of SAPs and fluff. The extent of mixing correlated with granule morphology: I3 contained smaller, smoother SAP granules than the larger, porous, angular SAP granules in I2, while the SAPs in I5 had intermediate morphologies. This could lead to differences in SAP mobility through fluff and therefore the stability of their distribution. Manufacturers may also choose double or multi-core structures to reduce leakage [58].

Drier top sheets and observed loss of sheen were associated with higher SAP loading and with coverstock material in the order PLA < cotton < viscose < PP. The product with PLA coverstock, I3, was unusual in that the top sheet contained an adhesive layer which may have presented a barrier to liquid flow-through, resulting in its high sheen despite a high SAP loading. For the remaining products, the above order correlates with single-layer vs. multilayer top sheets, increasing hydrophobicity of coverstock material, and increasing circularity of fibres, all of which may impact moisture transport. While apertured coverstock is reported to aid penetration and reduce rewet in the patent literature [56], results were mixed. In product M8, the apertured coverstock did demonstrate rapid strike through and apparent surface dryness. However, in product I7, liquid travelled both over and under the coverstock: on the top surface, liquid pooled at the point of insult for 5 s before rolling off. In contrast, non-apertured cotton products containing SAPs (I5–6) were observed to have fluid run-off from beneath the coverstock, which remained relatively dry. It may be that aperturing can aid strike-through but, in the absence of SAPs, fully cotton products hit a saturation point rapidly when high liquid volumes are dispensed.

While this simulation was informative of how moisture was distributed and of how products reached a saturation point under flow, it is limited in that products contour to the body during use and fluid run-off is therefore altered. Absorption before leakage measurements using a mannequin (NWSP 354.0.R2 (22) [75]) would better test structural leakage prevention in context [23]. Dosages also placed an emphasis on short-term absorption and single, rather than repeated, insults. As a result, the impact of SAPs on performance may be underrepresented; for example, I1 and I7 were products of a similar weight which absorbed similar amounts of fluid during testing, despite the lack of SAPs in I7. Future work could consider using Rothwell measurements (ISO 11948-1 [76]) as well to determine the maximum absorptive capacity, which correlates to leakage rates in use [23]. In this standard, products are weighed dry and then placed on wire mesh with the backsheet facing upwards. This is immersed into a vat of 0.9% saline for 30 min if the sample contains SAPs or 5 min if it does not. The mesh and sample are then removed from the vat and allowed to drain under gravity for 5 min, and the wet sample is weighed. The weight difference between dry and wet is the maximum absorptive capacity. This could better illustrate differences in the maximum amount of fluid different products can hold, largely uncoupled from product structure and allowing time for SAP expansion.

Two key factors in real use cases are not evaluated by the above measurements and should be considered for further work. Firstly, absorption efficiency decreases under pressure, leading to rewet and potential leakage under body weight during actions such as sitting. To assess this, rate of acquisition and rewet tests (NWSP 070.9.R1 (23) [77]) could give insight into how well liquid is retained under simulated pressure [23]. A single dose of 30 mL or 75 mL of 0.9% saline is dispensed onto an incontinence pad and allowed to fully absorb, as noted by the loss of wet sheen on the product surface; the time this takes is called the rate of absorbency. Ten minutes after full absorption, 10 g of dry filter paper is placed on top of the pad below a cylindrical weight exerting 1 psi pressure, both 9 cm diameter. After one minute, the filter paper is re-weighed and the difference between wet and dry weight is the rewet. This would allow for a comparison of expected product performances under human body weight. Secondly, users typically experience multiple voiding episodes. Both the simulated urination measurements and the rate of acquisition and rewet tests could be conducted using multiple rather than single doses to assess the effect on absorptive performance.

SAPs are expected to greatly exceed the performance of other typical absorbants in both maximum absorptive capacity and absorption under pressure [61]. Therefore, we would expect to see a greater difference in performance between commercial products with and without SAPs if further work were carried out. This highlights a challenge to production of highly absorbent, fully biodegradable products. Overall, the mass, thickness, and areal density of the top sheet layers made only a minor contribution to absorption in comparison to the absorbent core mass. While some structural aspects of top sheets were associated with reduced leakage, the absorbent core was key to absorption. In order to achieve the same performance, there is a clear need for alternatives to synthetic SAPs with comparative performance levels. There is a need for commercial biopolymer-based SAPs or for cellulosic foams, which may have potential to rival typical SAP performance.

### 3.3. Air Permeability

**Figure 9:**
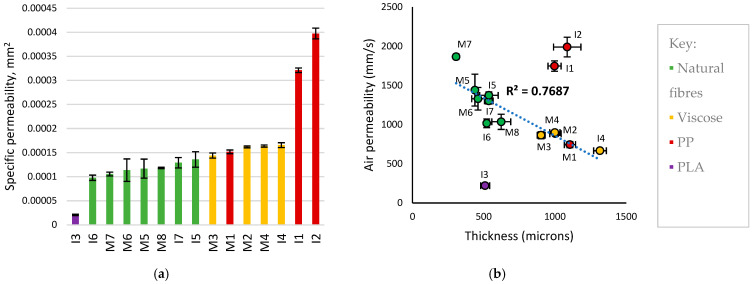
(**a**) Bar chart of specific permeability, ordered by increasing product mass. Dotted line separates products with an airlaid nonwoven transport layer from those without. The products are classified by the principal material of the coverstock; see Table 3 and Table 4 for a more detailed breakdown of materials. (**b**) Average air permeability as a function of top sheet thickness, with a linear trendline showing the relationship for products with cellulosic coverstock. Error bars show standard deviation.

As permeability of products can be key to user comfort and skin health [78], air permeability measurements were taken to assess gas transport properties. To account for differences in the thicknesses of product top sheets, Darcy’s law was used to calculate specific permeability, as shown in Figure 9a. When grouping products by the coverstock material employed, specific permeability increased in the order of PLA << natural fibres < viscose << polypropylene, which matched the trend in apparent drying rates of coverstock observed during absorption testing. In bulk materials, gas permeability increases with decreasing packing of polymer chains, and specific permeability would be expected to increase in the order cellulose < PLA < PE < PP as density, crystallinity, and polarity decrease [79]. However, in porous nonwovens, structural parameters such as packing density and average pore size make the significant contribution to permeability [80,81].

With the exception of I3, specific permeability increased from hydroentangled coverstocks (all cellulosics) to spunbond coverstocks (all thermoplastics), and from products that did not contain an airlaid nonwoven transport layer (PLA, natural fibres) to those that did (viscose, PP). Transport layers contained a polyolefin with either cotton (viscose, M1) or a thermoplastic (I1–2); the specific permeability increased as the ratio of synthetic to natural fibres in the entire top sheet increased. Increasing proportion of natural fibres and/or grooved viscose in nonwovens has been observed to reduce porosity: this has been attributed to a greater packing efficiency that arises from the higher surface area of noncircular fibres and the comparatively low stiffness of cellulosic fibres, leading to an overall reduction in specific permeability [82,83]. This has been observed in multilayer nonwovens as well as blends [84].

Permeability of nonwovens correlated with the bonding methods used, as previously reported [81], in the order bulk chemical < mechanical < thermal < point chemical or thermal. I3 was a PLA spunbond nonwoven which was chemically bonded to tissue paper. The wax-like adhesive layer presented a barrier to air flow, resulting in the lowest air permeability (221 mm·s^−1^) despite low areal density (50.2 g·m^−2^). Products with natural fibre coverstock universally consisted of hydroentangled (spunlace) nonwovens (average 1338 mm·s^−1^). Despite aperturing leading to greater apparent surface openness, this did not correlate with greater specific permeability. Products with hydroentangled viscose coverstock were paired with a cotton/PE airlaid nonwoven; as the airlaids were only bonded at thermoplastic fibre junctions, the materials retained comparatively high openness and large pore diameters (average 829 mm·s^−1^). I4 contained an additional odour-neutralising layer which reduced its permeability (667 mm·s^−1^). M1 had comparable air permeability to viscose products (745 mm·s^−1^) as its air permeability was dominated by its ADL rather than its thin PP coverstock. Lastly, I1–2 consisted of PP coverstock and fully synthetic airlaid ADLs. As the ADLs did not contain bulky natural fibres and were point-bonded, they retained high porosity and these top sheets had the highest air permeability (average 1868 mm·s^−1^).

Air permeability is plotted against thickness in Figure 9b. Both menstrual and incontinence products had similar average thickness and air permeabilities: 730 and 786 µm thickness, and 1133 and 1188 mm·s^−1^ permeability, respectively. In agreement with specific permeability data, air permeability of top sheets was grouped by coverstock material and their associated nonwoven structures. The secondary factor in determining air permeability was thickness: a linear relationship between air permeability and thickness was seen in top sheets with high cellulosic character, as path length through the material increased. A similar relationship was seen with areal density, where the air permeability of cellulosic top sheets decreased as areal density increased. No clear relationship was observed between the presence and size of apertures and the air permeability. I6 had unexpectedly low air permeability despite having similar thickness, areal density, and material to I5 and I7. This highlights that there are differences between products that may not be accounted for in this study; for example, surface finishing can lead to greater local packing density [80]. While the above air permeability data may suggest materials for better user comfort, the influence of top sheet breathability on incontinence-related dermatitis is not yet well-established. Existing studies highlight anti-microbial materials and microporous backsheets as potential product features to preserve skin health [78].

In this work, comfort thus far has been addressed through air permeability measurements and observations of surface sheen. Dry comfort of the top sheets is further explored through moisture management testing below. This is not a comprehensive study of comfort and further metrics exploring the tactile comfort of top sheets would be complementary for future work. Controlled sensory studies with human participants could be carried out to assess nonwoven or product feel, though costly. Mechanical analysis of materials may be performed to measure parameters relating to tactile softness: high surface smoothness, low friction, high flexibility, low stiffness, and high compressibility. Handle-O-Meter tests (which measure flexibility and friction as a function of resistance to the passage of the fabric sample through a slit) and friction, compression, and other mechanical testing with a Universal Testing Machine may be used to measure these properties. While some tests require isolated top sheets, it is of interest to know how the product functions as a whole. The Tactile Sensation Analyzer (emtec, Leipzig, Germany), which uses vibrational and mechanical testing to obtain material parameters, is one approach capable of comprehensive tactile testing of AHPs. Abrasion resistance measurements—such as the Martindale method [85], where a flat sample is rubbed against a standard abrasive material until failure, mass loss, or change in appearance—could also provide indirect insight into tactile comfort of nonwovens. Parameters associated with greater perceived softness are often also associated with lower abrasive resistance, and the durability and failure modes of the material may elucidate comfort during product wear over time.

### 3.4. Moisture Management Testing

**Figure 10:**
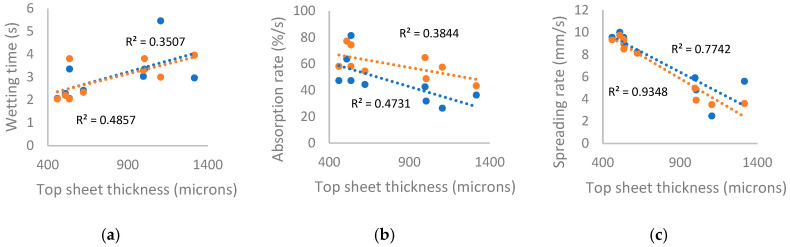
Top sheet thickness plotted against (**a**) wetting time; (**b**) absorption rate; and (**c**) spreading rate to show trendlines, excluding the data for I6. Data for the bottom surface is in orange, and top is blue. Linear trendlines are plotted as dotted lines.

The rate and directionality with which a top sheet transports fluid is key for user comfort and ensuring rapid distribution to the absorbent core. A subsection of products was characterised using MMT: six incontinence products (I1 and I3–7) and four menstrual products (M1–2, M6, and M8). This technique uses saline test fluid and was used to assess products in the context of incontinence. While saline can be used to model sweat and incontinence fluid management, this cannot be extrapolated to menstrual performance. Parameters for wicking at the top and bottom surfaces are shown in Figure 10 as a function of top sheet thickness. As expected, wetting times increased and absorption and spreading rates decreased as top sheet thickness increased, as the liquid travelled a longer distance in the Z axis. Top sheet thickness in the dataset was bimodal: top sheets with airlaid transport layers (viscose or PP coverstock) were thicker than those without (natural fibre or PLA coverstock). Greater differences between top and bottom surface absorption and spreading rate were seen as thickness increased; this is attributed to increased distance for fluid to travel and the presence of transport layers of a different material to the coverstock. Product I6 had an abnormally long bottom wetting time of 11 s compared to products with similar cotton coverstock, in addition to lower air permeability than expected; coverstocks may have surface treatments to promote softness or alter hydrophilicity, which may lead to differences in observed measurements [86]. Differences in dielectric constants for different fibres and surface finishes may also lead to perceived differences in wicking behaviour during measurement, as can residual adhesive or fluff pulp from pad dissection. It is pertinent to mention that the assessment was performed for top sheets only, and whole products might function differently given their multilayered structure.

A summary of MMT data is shown below in Table 7 alongside a description of the product top sheets. Excluding I6, the average top and bottom wetting times were similar between use cases, though menstrual products were associated with longer top wetting time: 3.32 and 2.79 s, and 2.74 and 3.06 s for menstrual and incontinence products, respectively. The average top and bottom absorption rate of menstrual products were slower at 37.5 and 54.7%/s, and 54.3 and 63.6%/s for menstrual and incontinence products, respectively. Likewise, the average top and bottom spreading rate were lower in menstrual products, with 6.27 and 6.22 mm·s^−1^, and 8.01 and 7.23 mm·s^−1^ for menstrual and incontinence products, respectively. These observations largely align with the relationships to thickness observed in Figure 10 above, as the incontinence products tested had thinner top sheets than menstrual on average (736.1 vs. 798.0 µm). The average thickness of incontinence top sheets further decreased to 644.7 µm when I4, which contained a charcoal-based odour-neutralising layer, was excluded.

**Figure 11:**
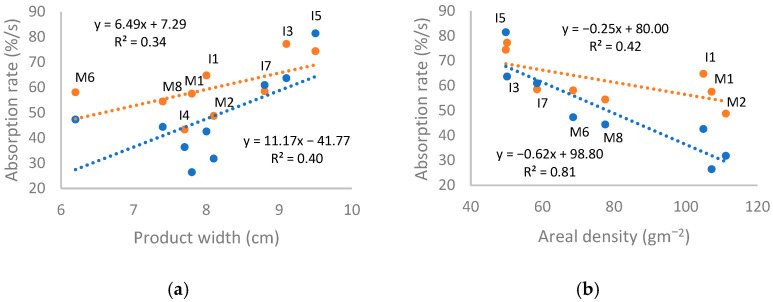
Absorption rates of bottom (orange) and top (blue) surfaces plotted against (**a**) product width and (**b**) areal density of top sheets. Linear trendlines are plotted as dotted lines. Areal density for I4 is not shown, as its top sheet contained a charcoal-based odour-control layer in addition to nonwovens.

A weak relationship was observed between absorption rates and product width, as shown in Figure 11a below, where wider products were associated with more rapid absorption at both top and bottom surfaces. Shorter products were similarly associated with higher bottom absorption rates, but no relation was seen for top surfaces (R^2^ values 0.28 and 0.00, respectively). No direct relationship to product length and width was seen for wetting times or spreading rates.

As shown in Figure 11b, negative correlations were observed between areal density of the top sheets and the absorption rates at surfaces. The correlation between areal density and bottom surface absorption rate is similar to that seen for thickness. As previous studies have found that areal density of coverstock has a negligible effect on absorption rate [16,87], this reflects a strong correlation between thickness and areal density in the dataset (R^2^ 0.80). However, differences in areal density in this dataset also correspond to differences in linear density of materials, differences in processing methods of nonwovens, and differences in multilayer structure, which may have significant impact.

Although little or no direct correlation was observed between product dimensions and top sheet thickness or areal density, the relations of product size to absorption rate can be rationalised as arising from product features associated with product dimensions. Within this dataset, products with core wrap structures were typically longer and thinner than products without (average length 27.8 and 24.9 cm, respectively). As the core wrap was used to reinforce the product, the average top sheets in these products had higher density and thickness (981.1 µm compared to 690.7 µm), while the products with lowest total areal density (I5 and I3) were relatively short. Meanwhile, incontinence products had higher average width than menstrual, to prevent leakage (7.4 and 8.6 cm, respectively). As stated above, incontinence products tested with MMT had lower thickness but also top sheet density than menstrual on average (644.7 vs. 798.0 µm, 65.9 vs. 91.1 g·m^−2^); this was observed even when the outlier I4 was included in the average (736.1 µm and 82.0 g·m^−2^). Products with similar nonwovens and structures tended to have similar dimensions, aligning with the trend for bottom absorption rate to increase from core wrap products to ADL products to single-layer products, with the exceptions of M6 and I7.

The correlation between areal density and top absorption rate was stronger than seen for thickness. This may be attributed to the structure or action of transport layers. With the exception of M6, width vs. top absorption rate is grouped into wide products with single-sheet top sheets (I3, I5, and I7) and narrower products with multilayer top sheets, which also correlates with areal density.

**Figure 12:**
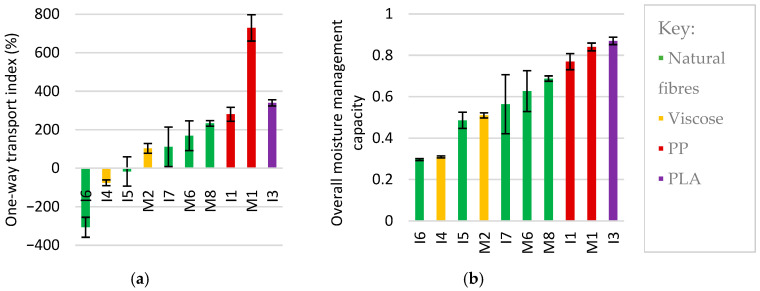
(**a**) Accumulative one-way transport index and (**b**) overall moisture management capacity (OMMC) of products, ordered by increasing OMMC. Error bars denote standard deviation.

Calculated performance parameters for each product are shown below. MMT compares the top and bottom surface parameters to give a one-way transport index for each top sheet, which indicates the expected wet back (Figure 12a). In contrast, the overall moisture management capacity (OMMC) is an overall assessment of a fabric’s ability to distribute moisture (Figure 12b). Product performance appeared grouped by cellulosic versus synthetic coverstock, rather than by AHP use case. The PLA top sheet (I3) had the highest OMMC, followed by products I1 and M1, which had PP coverstock and then by products with cotton and viscose coverstock. The high OMMC of I3 is unexpected: it had the lowest specific permeability of all products and was observed to have a high sheen on the top surface during absorption testing, implying poor liquid transport. Despite this, I3 had rapid wetting, absorption rate, and spreading rate. It appears that the adhesive layer was hydrophilic and water permeable, and the top sheet was wet effectively, leading to high sheen which did not readily dissipate.

Top sheets consisting of multiple nonwoven layers typically outperformed single-sheet coverstock in both metrics as water can be held in interstices between layers. M8, which had a bilayer cotton coverstock, outperformed all other cellulosic top sheets, while products with viscose coverstock (I4 and M2) were exceptions to this observation and had comparatively poor performance. The efficacy of one-way transport was related to the difference in surface energy of nonwovens: moisture transport may be driven from more hydrophobic materials to more hydrophilic materials by a push–pull effect [88]. Product M1 had approximately twice the one-way transport index of the next highest value measured, as the PP coverstock was much more hydrophobic than its cotton/synthetic ADL. Product I3 also had a large difference in surface energies and used PLA coverstock connected to a tissue core wrap by an adhesive layer to achieve rapid wetting and absorption. However, it was under half as thick as M1, meaning that its capacity to generate a moisture gradient was more limited. Compared to M1, products I1 and M8 had transport layers with more similar hydrophilicity to their coverstocks: I1 used PP in both coverstock and ADL, and M8 had bilayer cotton coverstock and therefore identical response. Lastly, products I4 and M2 had viscose coverstock, which was more hydrophilic than the cotton/PE core wrap and therefore resisted downward transport. I4 contained a deodorising layer, which further impeded flow. In addition, OMMC increased linearly from M2 to I1 to M1, in order of increasing difference in areal density of coverstock and transport layer (12.5, 52.7, and 66.2 g·m^−2^, respectively, where all have 107 ± 4 g·m^−2^ total; R^2^ value 0.9985). This may suggest that a finer coverstock and/or greater surface area in the transport layer enhances the efficiency and directionality of fluid transport; it is difficult to uncouple from potential differences in linear density of different materials used in these products.

While many biodegradable materials are hydrophilic, hydrophobic materials are desirable to generate the push–pull for improved one-way transport. PLA is a hydrophobic biopolymer and may present a viable alternative to polyolefins in coverstocks and airlaid transport layers. Surface treatments can be used to reduce the hydrophilicity/hygroscopic nature of cellulose nonwovens [86]; however, these may use nondegradable chemicals and coating reduces pore diameters, which may reduce permeability.

In products with natural fibre coverstock, one-way transport index and OMMC increased as the areal density increased, with the exception of I6. Previous studies have reported that areal density of coverstock has a negligible effect on the rate of wetting, spreading, and absorption [16,87]. In this work, there were significant differences in the structure of natural fibre coverstocks, which may better explain their performances. Product M6 contained fibres with a higher linear density than cotton, which implies a lower packing density than a cotton nonwoven of the same areal density. The use of aperturing in I7 and M8 coverstock to generate large-scale pores was associated with shorter wetting times compared to other cotton products I5–6, supporting observations made during absorption testing and in agreement with the patent literature, where aperturing was claimed to aid fluid penetration [56]. The bilayer structure in M8 increased porosity in that water could occupy interstices.

Due to the variety of materials in the products tested, fibre properties could not be directly related to wicking. Wetting is a prerequisite for wicking, and so hydrophobic coverstocks were associated with slow top surface spreading rate. Smaller fibre diameters generate greater capillary forces, and finer fibres have been seen to enhance wicking rate and absorption capacity [89]. This was not observed in natural fibre coverstock: product M6 had the coarsest natural fibres of coverstock tested, with an average diameter of 30.9 µm, yet the second highest wicking performance of the natural fibre top sheets. This is in agreement with reported absorption of artificial urine by cotton nonwovens, where finer cotton led to a slower absorption rate due to pore collapse [87]. As M6 consisted of a natural fibre and synthetic fibres, its behaviour may not result from natural fibre diameter alone. Lastly, fibre morphology may influence wicking. In products I4 and M2, the viscose fibres were observed to have longitudinal striations in SEM micrographs, which increases the rate of moisture transport by driving capillary wetting along channels on the fibre surface [90]. Despite this, these products were observed to have the second slowest spreading rates at the top surface: it is possible that the poor match in hydrophobicity caused moisture transport towards the interface between the two nonwoven layers in the top sheets. Nonetheless, bioplastic, regenerated cellulose, and natural fibres with ideal shape or morphology for wicking could be used in future nonwovens for absorbent hygiene applications.

As discussed in absorption testing, it should be noted that while the moisture management testing simulated moisture wicking under single doses, users often experience multiple voiding episodes. Further work should consider the impact of multiple doses using repeated liquid strike-through time measurements (NWSP 070.7.R2 (20) [91]). Coverstock or top sheets are placed on top of seven sheets of 100 × 100 mm blotter paper. This sample is positioned under a strike-through plate with a well and electrodes. A total of 5 mL of 0.9% saline is dispensed into the well, and the time to drain is detected conductometrically and recorded as strike-through time. The next dose is dispensed one minute after the previous strike-through. This could be further combined with wetback measurements (NWSP 070.8.R1 (19) [92]), where a 4 kg weight and dry paper are placed on the sample following strike-through tests and the weight difference between dry and wet paper is recorded as the wetback. This would give insight into the absorbent performance of the nonwovens under pressure from the human body.

A summary of the design, and structural and material construction of products is given below in Table 8 with observed performance metrics. Overall, a wide range of designs and properties were observed both within and between use cases.

## 4. Conclusions

To the authors’ knowledge, this is the first public analytical study comparing the design and components of commercial absorbent hygiene products. Products were deconstructed, analysed, and assessed to gain holistic insight into how materials and product design lead to performance. A broad range of materials and product constructions were observed. While market-leading products made greater use of petrochemically derived polymers, natural fibres, regenerated cellulose, and PLA were observed as alternatives to polyolefin nonwovens, which were marketed as more sustainable. Biodegradable polymers containing PBAT, PLA, and/or starch were observed as sustainable alternatives to polyethylene films and appeared to be relatively well-established, present in two-thirds of products. Another product made use of a biologically derived and oxodegradable polyethylene backsheet; however, this approach is challenged by both lower production and higher cost of biologically derived PE and the legislation and lack of consensus regarding the environmental impact of oxodegradation. However, two-thirds of products used superabsorbent polymers, and there does not appear to be a fully biodegradable alternative on the market. Products without superabsorbent polymers used cellulosic materials in their absorbent core (cellulosic fluff, card wadding, and/or carboxymethylcellulose hydrogel), but these are unable to achieve the same absorption efficiencies as superabsorbent polymers, particularly under pressure. The development of biodegradable SAP alternatives is a considerable challenge for the development of sustainable products with high performance. Future candidates may include biopolymer-based SAPs and/or cellulosic foams.

User comfort parameters of the top sheets, i.e., air permeability and moisture management testing, were linked to material choice and/or nonwoven structure. Coverstock materials were associated with specific top sheet structures: products with regenerated cellulose or PP coverstock all contained airlaid or spunbond moisture transport layers, and the product with a PLA coverstock contained a water-permeable layer with restricted air flow. Permeability increased depending on nonwoven bonding in the following order: Chemical < Mechanical < Thermal < Point chemical or thermal. Synthetic polymers typically outperformed cellulosics in both metrics. Top sheets with spunbond PP coverstock had high intrinsic permeability and overall moisture management capacity, top sheets with hydroentangled natural fibres and/or regenerated cellulose coverstocks had intermediate permeability and low or intermediate OMMC, while spunbond PLA coverstock had the lowest intrinsic permeability but the highest OMMC. Lower thickness and areal density of top sheets was associated with higher air permeability. However, greater thickness and areal density was seen to be advantageous for wicking and one-way moisture transport. Aperturing of top sheets had little effect on air permeability but appeared to shorten wetting and penetration time in absorption testing and MMT. Layered top sheets, where the moisture transport layer had hydrophilicity equal to or greater than the coverstock material, were seen to have improved moisture transport metrics and one-way moisture transport during absorption testing and MMT. Layered structures increase porosity in that water can occupy interstices, and a greater difference in hydrophilicity between layers was observed to increasingly drive fluid to the hydrophilic material via a push–pull effect. This effect could be leveraged to reduce strike-through time and keep coverstock drier in use. Further work could explore tactile comfort to assess perceived softness and explore repeated wicking to assess performance in the case of multiple voiding events.

There were clear differences in design between menstrual and incontinence products, which likely relate to differences in expected fluid volume. Menstrual products had thinner, lighter pads and favoured designs with embossing; wings; core wraps; no fluff; and layered SAPs. Incontinence products had thicker, heavier pads and favoured side strips; lateral elastic; no wings; ADLs or no moisture transport layer; fluff; and blended SAPs. These strategies were used to shape products and prevent leakage with the lowest achievable thinness, as appropriate for the use case. Channels, side strips, and lateral elastic appeared to prevent side leakage and improve distribution of fluid throughout the entire product core during simulated urination tests. These features may help prevent leakage and ensure maximum performance from all materials used. However, a key limitation of this study is that products were tested flat. The effect of product shaping could be further explored with testing of products worn by a mannequin. Although product structure and top sheets appeared to contribute to strike-through and fluid distribution, the overall absorbency of products correlated with absorbent core mass and higher SAP loading was associated with improved fluid distribution and drier coverstock. During product use, SAPs are further expected to greatly reduce rewet under pressure. Further work to quantify the mass and absorption capacity of SAPs in AHPs, to collect full product absorption capacities, and to perform rewet testing would give greater insight into product design for more efficient use of absorbent materials. Further work with multiple dosages would also give insight into product performance in use, as it is common for users to experience multiple voiding episodes. While this work focused on management of incontinence, similar work could be carried out to assess product performance for absorption of menses; an appropriate test fluid must be used to assess their capacity for management of menstruation.

Overall, while commercial products are beginning to embrace sustainable materials, there remains an urgent need for biodegradable SAPs in order to achieve comparable performance to products with polyacrylic acid-based SAPs for the management of large fluid volumes. However, prudent product design was able to enable products without SAPs to be effective for the management of smaller fluid volumes, despite the difference in intrinsic absorbency between cellulosics and SAPs. We hope that this study will inform the future design and material selection of sustainable absorbent hygiene products.

## Figures and Tables

**Table 1:** Artificial urine formulation, showing the ISO 20696 formulation, the reported midpoint values of human urine composition [30], and the final artificial urine formulation used in this work.

Chemical	Concentration (g·L^−1^)		
	ISO 20696	Human	Artificial
Urea	25.0	16.3	16.67
Sodium chloride	9.0	-	6.00
Ammonium chloride	3.0	-	2.00
Disodium hydrogen orthophosphate	2.5	-	1.67
Potassium dihydrogen orthophosphate	2.5	-	1.67
Creatinine	2.0	1.64	1.33
Sodium sulphite (hydrate) *	2.0 (3.0)	-	1.00 (2.00)
Sodium ion	4.9	2.78	3.27
Potassium ion	0.72	1.68	0.48
Chloride ion	7.4	5.14	4.93

* ISO 20696 lists the hydrated form of sodium sulphite in its list of components.

**Table 2:** Summary of product construction and components for the selected products; incontinence products are labelled I1–7 and menstrual M1–8. Product features are either specified, denoted with Y for yes if present, or cells are left blank to indicate that the feature is absent in that product. ADL and CW are abbreviations for acquisition distribution layer and core wrap, respectively. Product weights taken prior to disassembly.

	Dimensions (cm)			Mass	Transport	Absorbent Core			Other Features	
	Length	Width	Height	(g)	Layer	Fluff	Granules	Wadding	Wings	Side Guards
I1	23.9	8.0	0.65	9.945	ADL	Y	Y			
I2	39.1	12.1	1.4	35.434	ADL	Y	Y			Elastic
I3	23.0	9.1	0.42	7.566	CW	Y	Y			Strips
I4	27.5	7.7	0.32	8.856	CW		Y	Y	Y	Elastic and strips
I5	25.0	9.5	-	14.637		Y	Y	Y		Elastic
I6	32.0	12.0	0.90	24.239		Y	Y	Y		Elastic
I7	30.8	8.8	0.60	10.789		Y		Y		
M1	23.6	7.8	0.27	4.964	ADL	Y	Y		Y	
M2	28.0	8.1	0.25	6.719	CW		Y	Y	Y	
M3	27.9	7.8	0.09	7.136	CW		Y	Y	Y	
M4	28.0	7.7	0.10	7.272	CW		Y	Y	Y	
M5	27.5	7.5	0.04	8.769	CW				Y	
M6	23.0	6.2	0.36	8.095		Y	Y		Y	
M7	25.5	7.3	0.03	7.050	CW	Y			Y	
M8	27.9	7.4	0.35	7.101	CW	Y			Y	

**Table 3:** Summary of coverstock and total top sheets in the commercial AHPs. Products which contain transport layers are marked by italics. H stands for hydroentangled nonwovens, with a designation of *s*, *l* or *a* for finishes that are smooth, have visible linear rows of jetting pores, or are apertured. SB, A, and C refer to spunbond, airlaid, and carded nonwovens, divided into bulk bonding *b* and point bonding *p*. Embossing is indicated with Y for yes if present and cells are left blank if absent.

	Coverstock				Total Top Sheets			
	Material	Mass (g)	Density (g·m^−2^)	Fibre Diameter (µm)	Thickness (µm)	Mass (g)	Density (g·m^−2^)	Embossing
*I1*	PP, SB_p_	0.444	26.13	18.79	997	1.371	104.93	
*I2*	PP, SB_p_	1.258	27.07	21.21	1086	1.816	92.64	
*I3* *	PLA, SB_p_	0.492 *	50.16	17.50	510	0.492	50.16	Y
*I4* #	Viscose, H_s_	0.888	45.46	15.80	1317	3.221 #	171.14 #	Y
I5	Cotton, H_l_	0.924	49.82	23.62	536	0.924	49.82	
I6	Cotton, H_l_	1.431	54.67	21.54	521	1.431	54.67	
I7	Cotton, H_a_	1.043	58.52	21.69	536	1.043	58.52	
*M1*	PP, SB_p_	0.364	20.55	18.10	1105	1.547	107.25	Y
*M2*	Viscose, H_s_	0.899	59.69	13.80	1004	2.682	111.22	Y
*M3*	Viscose, H_s_	0.916	37.54	12.45	903	2.940	91.99	Y
*M4*	Viscose/cotton, H_s_	0.923	42.06	12.91/26.37	1000	3.016	98.66	Y
*M5* †	Hemp/viscose, H_a_	1.351	59.30	-/15.21	438	1.351	59.30	Y
M6 ‡	Natural fibre/‡, H_l_	1.109	68.57	30.92/13.46	460	1.109	68.57	
*M7* *	Cotton, H_a_	0.858	41.96	16.65	307	0.858	41.96	Y
*M8* ⸸	Cotton, H_a_	0.804 ⸸	77.55 ⸸	21.92	623	2.245	77.55	Y

Products which contain transport layers are marked by italics. * I3 and M7 had tissue paper core wraps. In I3, this was bonded to the nonwoven coverstock and to the backsheet with unidentified waxy material. In M7, tissue was removed from top sheets for tests. # I4 contained a charcoal-based deoderising layer; this was between nonwovens and remained in place for characterisation of the product’s top sheets. † M5 was unusual in that a folded nonwoven acted as the absorbent core without any of the other typical absorbent components. The coverstock alone is taken to be the top sheet in testing. ‡ M6 contained smooth filaments or fibres that were masked by natural fibre in the FTIR spectra. This fibre was soluble and was isolated via filtration. The soluble fibre FTIR spectrum bore some resemblance to poly vinyl alcohol (PVA), poly vinyl pyrrolidone (PVP), and poly acrylamide (PAM) but could not be definitively identified. ⸸ M8 had a bilayered coverstock which acted as both coverstock and core wrap. It was excluded from average values for coverstock and core wrap materials for this reason.

**Table 4:** Summary of moisture transport layers in commercial AHPs. Products which contain ADLs are marked with italics, while others contain core wraps.

	Material	Mass (g)	Density (g·m^−2^)	Fibre Diameter (µm)
*I1*	PP/*, A_p_	0.927	78.80	23.64 *
*I2*	PP/*, A_p_	0.558	65.57	23.10 *
*M1*	Cotton/#, A_b_	1.183	86.70	39.37/14.42
I3	Tissue paper	-	-	-
I4	Cotton/PE, A_b_	2.185	57.97	30.43/15.52
M2	Cotton/PE, A_b_	1.783	51.53	30.58/14.65
M3	Cotton/PE, A_b_	2.024	54.45	32.12/19.66
M4	Cotton/PE, A_b_	2.093	56.59	28.03/18.03
M5 †	Hemp, C_b_	5.387 †	-	-
M7 ‡	Tissue paper	0.907	-	-
M8 ⸸	⸸ Cotton, H_a_	1.441	77.55 ⸸	23.76

Products which contain ADLs are marked with italics, while others contain core wraps. * I1 and I2 were found to contain a polyester or polyacrylate, the precise identity of which was not determined. # M1 contained a nonwoven with unidentified smooth filaments or fibres that were masked by natural fibre in the FTIR spectra. † M5 was unusual in that a folded nonwoven acted as the absorbent core without any of the other typical absorbent components. The coverstock alone is taken to be the top sheet in testing. The core layer consisted of fibre bundles of variable size. ‡ M7 had a tissue paper core wrap to contain fluff pulp, which was removed for testing of top sheets. ⸸ M8 had a bilayered nonwoven which acted as both coverstock and core wrap. It was excluded from average values for coverstock and core wrap materials for this reason.

**Table 5:** Summary of absorbants in the commercial AHPs. Morphology and average particle Feret diameter are given where available, with standard deviation. Absorbants of each subclass are either specified, denoted with Y for yes if present, or cells are left blank to indicate their absence.

	Absorbants				SAPs		
	Fluff	Granules	Wadding	Total Mass (g)	Distribution	Morphology	Feret Diameter (µm)
I1	Y	SAP		7.535	Blended	Jagged	860.14 ± 225.70
I2	Y	SAP		30.603	Blended	Jagged	
I3	Y	SAP		4.262	Blended	Cluster of spheres	632.17 ± 114.43
I4		SAP	Y	3.567	Layered	Cluster of spheres	
I5	Y	SAP	Y	11.404	Layered		
I6	Y	SAP	Y	19.408	Layered	Mixed	615.38 ± 174.97
I7	Y		Y	7.897			
M1	Y	SAP		2.351	Blended		
M2		SAP	Y	2.383	Layered	Cluster of spheres	467.98 ± 93.27
M3		SAP	Y	2.651	Layered	Cluster of spheres	551.14 ± 138.65
M4		SAP	Y	2.502	Layered	Cluster of spheres	475.56 ± 113.59
M5 *				5.387			
M6	Y	CMC		3.387			
M7	Y			3.542			
M8	Y			3.022			

* M5 was unusual in that a folded nonwoven acted as the absorbent core without any of the other typical absorbent components. It was excluded from calculations of average product compositions.

**Table 6:** Summary of backsheets in commercial products. Specific PBAT polymer blends are suggested.

Incontinence Products			Menstrual Products		
	Material	Mass (g)		Material	Mass (g)
I1	PE	1.208	M1	PE	0.715
I2	PE-CaCO_3_	2.243	M2	PLA/PBAT	1.057
I3	PLA/PBAT/starch	1.987	M3	Oxo-degradable PE-CaCO_3_	0.907
I4	PLA/PBAT	0.913	M4	PLA/PBAT	1.077
I5	PE-CaCO_3_	1.336	M5	PLA/PBAT/starch	1.027
I6	PBAT/starch	1.481	M6	Starch-based film	2.356
I7	PBAT/starch	1.183	M7	PLA/PBAT/starch	1.025
			M8	PLA/PBAT/starch	1.117

**Table 7:** MMT data and product top sheet structure.

		Wetting Time (s)		Absorption Rate (%/s)		Spreading Rate (mm·s^−1^)	
	Top Sheets	Top	Bottom	Top	Bottom	Top	Bottom
I1	Single (SB_p_ PP) A_p_ synthetic ADL	3.03	3.29	42.6	64.8	5.91	4.97
I3	Single (SB_p_ PLA) Tissue paper CW w/film	2.28	2.20	63.7	77.2	10.00	9.73
I4	Single (H_s_ viscose) Charcoal deodorant A_b_ cotton/PE CW	2.96	3.96	36.4	43.4	5.60	3.60
I5	Single (H_l_ cotton)	3.35	3.80	81.4	74.4	9.00	8.51
I6	Single (H_l_ cotton)	2.63	11.00	41.8	26.9	9.88	6.66
I7	Single (H_a_ cotton)	2.07	2.04	47.3	58.1	9.55	9.35
M1	Single (SB_p_ PP) A_b_ cotton/synthetic, ADL	5.46	3.00	26.5	57.5	2.48	3.50
M2	Single (H_s_ viscose) A_b_ cotton/PE CW	3.35	3.81	31.8	48.8	4.83	3.90
M6	Single (H_l_ natural fibre/soluble fibre)	2.07	2.04	47.3	58.1	9.55	9.35
M8	Double (H_a_ cotton): acts as coverstock and CW	2.42	2.33	44.4	54.4	8.21	8.12

**Table 8:** Summary of commercial product design and performance metrics.

	Top Sheets	Features	Absorbent Core	Absorption (g/g)	OMMC	Permeability (mm·s^−1^)
I1	Single (SB_p_ PP) A_p_ synthetic ADL		Fluff blended SAPs (jagged)	94.1	0.770	1747
I2	Single (SB_p_ PP) A_p_ synthetic ADL	Elastic Side strips	Fluff blended SAPs (jagged), double core	240.6		1990
I3	Single (SB_p_ PLA) Tissue paper CW w/film	Embossing Elastic Side strips	Fluff blended SAPs (clustered spheres)	86.0	0.870	221
I4	Single (H_s_ viscose) Charcoal deodorant A_b_ cotton/PE CW	Wings Embossing Elastic	Wadding layered SAPs (clustered spheres)	84.5	0.309	667
I5	Single (H_l_ cotton)	Elastic	Fluff, wadding layered SAPs	172.7	0.486	1373
I6	Single (H_l_ cotton)	Elastic	Fluff, wadding layered SAPs (mixed)	221.0	0.297	1017
I7	Single (H_a_ cotton)		Fluff, wadding	96.3	0.564	1303
M1	Single (SB_p_ PP) A_b_ cotton/synthetic, ADL	Wings Embossing	Fluff blended SAPs	38.5	0.841	745
M2	Single (H_s_ viscose) A_b_ cotton/PE CW	Wings Embossing	Wadding layered SAPs (clustered spheres)	49.4	0.510	884
M3	Single (H_s_ viscose) A_b_ cotton/PE CW	Wings Embossing	Wadding layered SAPs (clustered spheres)	46.1		865
M4	Single (H_s_ viscose/cotton) A_b_ cotton/PE CW	Wings Embossing	Wadding layered SAPs (clustered spheres)	47.7		899
M5	Single (H_a_ hemp/viscose)	Wings Embossing	C_b_ Hemp	30.0		1440
M6	Single (H_l_ natural fibre/soluble fibre)		Fluff blended CMC	43.3	0.627	1330
M7	Single (H_a_ cotton) Tissue paper CW	Wings Embossing	Fluff	45.7		1867
M8	Double (H_a_ cotton): acts as coverstock and CW	Wings Embossing	Fluff	50.0	0.688	1036

## Data Availability

The raw data supporting the conclusions of this article will be made available by the authors on request.

## Abbreviations

The following abbreviations are used in this manuscript:

ADL	Acquisition/distribution layer
AHP	Absorbent hygiene product
Bio-PE	Biomass-derived polyethylene
CMC	Carboxymethylcellulose
CNF	Cellulose nanofiber
CW	Core wrap
EDANA	European Disposables and Nonwovens Association
FTIR	Fourier transform infrared (spectroscopy)
FTIR-ATR	Fourier transform infrared attenuated total reflectance (spectroscopy)
MMT	Moisture management testing
NaCMC	Sodium carboxymethylcellulose
NaPAA	Sodium polyacrylic acid
NMMO	N-Methylmorpholine N-oxide
OMMC	Overall moisture management coefficient
PAA	Polyacrylic acid
PAM	Polyacrylamide
PBAT	Polybutylene adipate terephthalate
PBS	Polybutylene succinate
PCL	Polycaprolactone
PE	Polyethylene
PEA	Polyester amide
PHA	Polyhydroxyalkanoate
PHBV	Poly(3-hydroxybutyrate-co-3-hydroxyvalerate
PLA	Polylactic acid
PP	Polypropylene
PVA	Polyvinyl alcohol
PVP	Polyvinyl pyrrolidone
SAP	Superabsorbent polymer
SB	Spunbond
SEM	Scanning electron microscopy
